# Printing cell-laden gelatin constructs by free-form fabrication and enzymatic protein crosslinking

**DOI:** 10.1007/s10544-014-9915-8

**Published:** 2015-02-01

**Authors:** Scott A. Irvine, Animesh Agrawal, Bae Hoon Lee, Hui Yee Chua, Kok Yao Low, Boon Chong Lau, Marcelle Machluf, Subbu Venkatraman

**Affiliations:** 1 Materials and Science Engineering, Division of Materials Technology, Nanyang Technological University, N4.1-01-30, 50 Nanyang Ave, Singapore, 639798 Singapore; 2 Faculty of Biotechnology and Food Engineering, Technion University, Haifa, Israel

**Keywords:** Free form fabrication, Bioink, Transglutaminase, 3-D printing, Gelatin, Hydrogel

## Abstract

Considerable interest has arisen in precision fabrication of cell bearing scaffolds and structures by free form fabrication. Gelatin is an ideal material for creating cell entrapping constructs, yet its application in free form fabrication remains challenging. We demonstrate the use of gelatin, crosslinked with microbial transglutaminase (mTgase), as a material to print cell bearing hydrogels for both 2-dimensional (2-D) precision patterns and 3-dimensional (3-D) constructs. The precision patterning was attained with 3 % gelatin and 2 % high molecular weight poly (ethylene oxide) (PEO) whereas 3-D constructs were obtained using a 5 % gelatin solution. These hydrogels, referred to as “bioinks” supported entrapped cell growth, allowing cell spreading and proliferation for both HEK293 cells and Human Umbilical Vein Endothelial Cells (HUVECs). These bioinks were shown to be dispensable by robotic precision, forming patterns and constructs that were insoluble and of suitable stiffness to endure post gelation handling. The two bioinks were further characterized for fabrication parameters and mechanical properties.

**Electronic supplementary material:**

The online version of this article (doi:10.1007/s10544-014-9915-8) contains supplementary material, which is available to authorized users.

## Introduction

Bioprinting of tissue engineered scaffolds for regenerative medicine by Freeform Fabrication (FFF) allows for considerable architectural control for both 2-D and 3-D designs (Hollister 2005; Billiet et al 2012). Furthermore, there is a recognized potential for cells to be incorporated into the fabrication process, as a “bottoms-up” approach with the advantage of manufacturing a construct with an even distribution of viable cells. Techniques such as electrospinning have previously been developed to deliver cells directly into a construct (Ang et al 2014), however this technique suffers from a lack of deposition control due to the randomness in electrospun fiber grounding. FFF offers the potential of precision fabrication using cell bearing material under computer control. Materials for FFF of cell loaded structure are limited by the need for cytocompatability, however it is not possible to use common synthetic biocompatible polymers, such as polycaprolactone (Hutmacher et al. 2001), poly L-lactic acid (Yang et al. 2004) and polyurethane (Heijkants et al. 2004), due to their cytotoxic solvents. FFF scaffolds synthesized from these polymers only allow for post-fabrication cell seeding, resulting in uneven cellular distribution (Liu and Bhatia 2002; Martin et al. 2004; Stephens et al. 2007). Whereas water soluble hydrogel systems can deliver cells evenly within a cytocompatible and “tissue mimicking” environment ideal for bioengineering soft tissues (Billiet et al. 2014; Underhill et al. 2012; Fedorovich et al. 2011; Peppas 1997). Moreover gelatin hydrogels in particular are inexpensive to fabricate and are formed with relative ease.

For a polymer solution to be printable in 2-D, the viscosity has to be sufficient to produce distinct trace pattern that retains integrity prior to the completion of crosslinking. Furthermore, to build up in 3-D, the deposited hydrogel must be able to support the weight of the emerging structure without collapse. In previous examples, the gelatin deposition was carried out by heating the gelatin solution beyond physiological temperature (i.e., ≥40 °C) followed by delivery into a cold environment or by modifying the gelatin with photo-cross-linkable polymer; only the latter being cytocompatible (Peppas 1997; Liu et al. 2013; Yoo and Polio 2010; Nichol et al. 2010; Seliktar 2012) Here, we employ enzymatic crosslinking, which is seen as less likely to have unwanted side reactions (due to substrate specificity) and cell toxicity. Even though less cytotoxic photo-crosslinkers have been formulated (Mironi-Harpaz et al. 2012), the use of enzymes circumvents the need for specialized equipment and photo-sensitive additives. Indeed, Teixeira et al. (2012) reviewed the advantages offered by enzyme crosslinking, which include: relatively mild reaction conditions such as neutral pH, aqueous medium and physiological temperature; and the natural occurrence of some enzymes such as transglutaminase (Tgase). Tgase catalyses the bond formation between the γ-carbonyl group of glutamine residue and the ε-amino group of a lysine residue (Greenberg et al 1991; Collighan and Griffin 2009). Gelatin contains 8.4 % glutamine and 2.9 % lysine residues hence a suitable substrate for Tgase (Crescenzi et al. 2002). Ito et al. (2003) incorporated NIH/3T3 fibroblasts into Tgase crosslinked gelatin enzyme-crossslinked gel with the cells remaining viable for 1 week.

Free form fabrication has been shown to effectively create gelatin based scaffolds by indirect fabrication (He et al. 2008; Tan et al. 2010). However the processing required for these scaffolds limits these techniques to post fabrication seeding. In this work, we report on the development of “bioinks”, printable gelatin hydrogels that encapsulates viable cells and are extrudable into patterned constructs via using rapid prototyping. We optimized two gelatin bioink systems for bioengineering, firstly a 2-D bioink for precision deposition of a cell containing hydrogel traces without ink bleeding and a 3-D bioink, for building up in the z-axis. The 2-D bioink comprised of 3 % gelatin, and 2 % polyethylene oxide (PEO) (as a thickener) in PBS whereas the 3-D printing bioink is contains 5 % gelatin in PBS. We demonstrate their printability, entrapped cell viability (for both endothelial cells (HUVECs) and Human Embryonic Kidney (HEK) 293 cells) and hydrogel properties were characterized.

## Methods

### Materials

Gelatin from porcine skin (Bloom 300, type A) and polyethylene oxide (PEO) MW 600 K and phosphate buffer saline (PBS) pH 7.2 were purchased from Sigma-Aldrich. The mTgase (TG-BW-MH, 100U/gram, EC 2.3.2.13) with sodium casinate and maltodextrin additives was purchased from Ajinomoto (Japan). GFP (Green fluorescence protein) HUVECs were kindly donated by Prof. Gera Neufeld (Technion, Faculty of medicine, Israel), and GFP HEK 293 cells were obtained from Cell Biolabs, USA. Endothelial growth media (EGM-2) with supplements was supplied by Lonza and MEM media for HEK293 cells, fetal bovine serum (FBS) and penicillin streptomycin solution were purchased from Invitrogen.

### Preparation of cell containing gelatin mTgase bioink

Bioink solutions were prepared by dissolving gelatin at 3 % (with/without 2%PEO), 5 and 7 % (*w*/*v*) in 1xPBS buffer solution. The solutions were heated and stirred at 60 °C for 2 h to aid solubilization.

HUVECs and HEK293s were prepared as a cell suspension of 5 × 10^6^ cells ml^−1^ within the gelatin bioinks. Then mTgase preparation was added at concentrations of 0.5, 1.5 or 3 U/ml.

### Cell viability

The bioinks were extruded into thin lines via 30 gauge needle onto a 10 cm diameter non cell adhering polystyrene culture dish. After extrusion, samples were incubated at 37 °C for 30 min for cross linking to proceed. Subsequently, 10 ml growth media was added and replaced every 48 h throughout the experimental process. Cell viability was assessed by observing and recording the presence and density of GFP positive cells by fluorescence microscopy for up to 7 days.

### Robotic dispensing system

**Fig. 1:**
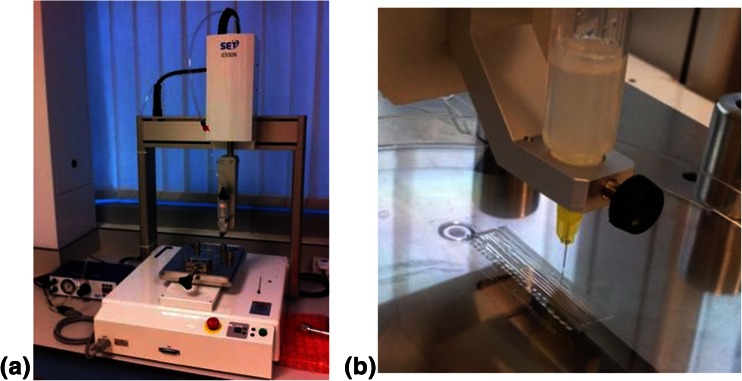
Freeform fabrication apparatus. The setup consists of a three axis XYZ movable robotic system controlled by a computer and a pressure driven syringe dispenser to dispense the ink (**a**). Close up of dispensing table the printing structure was 4 cm long along the printing direction (**b**)

The Janome 2300N pressure controlled robotic dispensing system consists of a computer control robotic XYZ table and a pressure driven syringe mechanism (Fig. 1). The software controls XYZ geometry and deposition rate. Printing was performed using either 0.05 or 0.1 MPa back-pressure, 5 mm/s writing speed, from a 25 ml syringe containing and 30 gauge needle (inner diameter 250 μm). *For the action view* supplementary video file.

### Rheological analysis of bioink gelation

The bioink solutions were cross linked with several mTgase concentrations in the absence of cells Anton Paar MCR 501 rheometer was used to calculate the viscosities, gelation time and gel shear storage modulus (G′) for the different bioink formulations at 37 °C. The testing was performed at 10 Pa and 1Hz frequency. The gel point for the crosslinking reactions were measured by recording the time at which the storage shear modulus overtakes the loss shear modulus. The reported value assumes the imaginary component (loss shear modulus G″) was negligible (G^*^ = G′ + iG″) (Mironi-Harpaz et al. 2012). In addition, the viscosities of gel solutions were measured at different temperature (20–40 °C) by heat-cool cycle.

### Scanning electron microscope (SEM) of porous gel network

For the SEM imaging JOEL 6360 was used, lyophilized dried hydrogel sample were sputter coated with gold for 30 s prior to imaging. Hydrogels of 5 % gelatin gel and 3 % gelatin /2 % PEO gel with 3U/ml mTgase were prepared and lyophilized. These gels were used in SEM with and without prior dialysis in deionized water for 1 week.

### AlamarBlue® assay for cell viability

Cell laden hydrogels, both printed and non printed, were placed in separate wells of a 24-well plate. Then 0.5 ml of 10 % AlamarBlue® solution was added. Reaction was then incubated in darkness for 4 h before analysis. After incubation, four 100 μl replicates were taken from each well and transferred to a black 96-well plate for fluorescence reading by a Varioskan flash reader (Thermo Fisher Scientific, MA, USA) (excitation 540 nm, emission 585 nm). Cell viability was calculated from the fluorescence relative to the control.

## Results and discussion

### Gelatin compositions

For this study Type A (acid extracted) gelatin with a bloom of 300 (average molecular weight 90 kDa) was used as this type has the greatest viscosity and intact glutamine residues (compared to the alkali extracted Type B gelatin forms). The enzyme chosen microbial transglutaminse (mTgase) rather than mammalian forms as it is not calcium dependent and has more robust enzymatic action (Bertoni et al. 2006). The construction of cell bearing scaffolds with gelatin methacrylate in several similar studies used gelatin concentrations of 10 % *w*/*v* and greater. These concentrations confer for suitable strength and load bearing characteristics to build up 3D structures. However it has been observed on gelatin methacrylate (GelMA) hydrogels that reductions in the gelatin concentrations from 15 to 10 to 5 %, conferred for increased HUVEC viability (Nichol et al. 2010). The lower concentrations of gelatin provide increased porosity and a more compliant gel to allow greater cell spreading, hence we investigated the possibility of using lower gelatin concentration hydrogels for cell seeding at 3, 5 and 7 %, although the 3 % gelatin could not retain its printed structure so that the inclusion of 2 % polyethylene oxide as a viscosity additive was required. As expected, it was observed that HUVECs were more able to spread and form pseudopodia in gelatin hydrogels of 3 % gelatin/2%PEO and 5 % gelatin whereas at 7 % gelatin the cells remain more rounded (Figure S1). Therefore the gelatin hydrogels will be used at the lower range of gelatin concentrations (3 to 5 %) for the entrapped cells to spread and fully express their phenotype.

### Assessment of Bioinks for 2-D precision printing at 37 °C

**Fig. 2:**
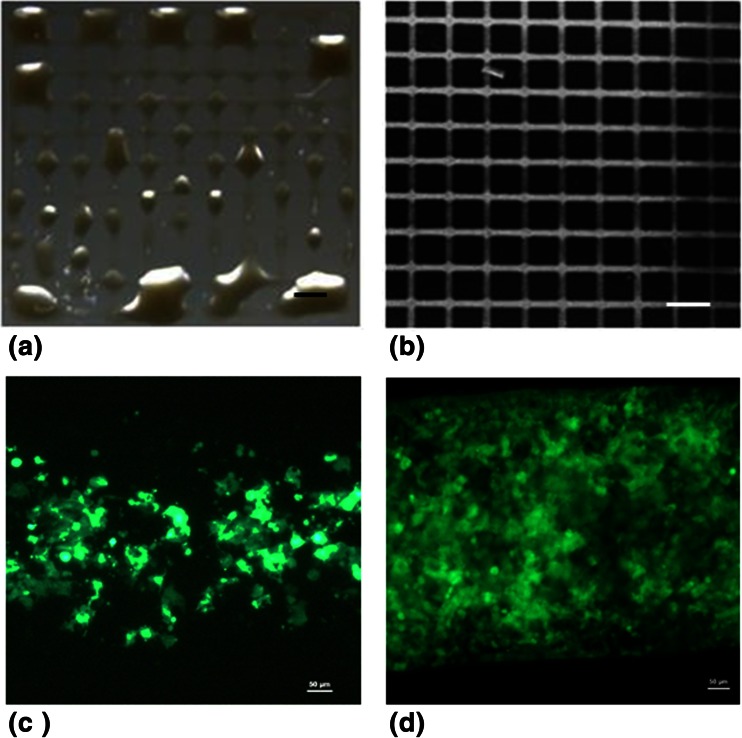
2D Bioink printing with HEK293 cells. A defined grid structure was deposited by FFF method, for the bleeding prone 5 % gelatin bioink (**a**) and the bleed free 3 % gelatin/2%PEO 2-D bioink. **b**. The drawing speed was 8 mm/cm and back pressure of 0.05 MPa. Scale bar = 2 mm. HEK293 cells were deposited in a 3 % gelatin/2%PEO bioink, viewed on day 1 (**c**) and day 6 (**d**). Images viewed by fluorescence microscopy using FITC (460–490 nm) filter, Scale bar = 100 μm

To observe the definition of the printed hydrogels, the robotic dispensing system was loaded with pre-warmed (37 °C optimal temperature for cellular metabolism) 3 % gelatin/ 2 % PEO or 5 % gelatin, and a grid printing programme was performed with 0.1 MPa through a fine gauge 150 μm diameter needle. The deposited 5 % gelatin-hydrogels were prone to ink bleeding and inconsistent printing (Fig. 2a), the grid lines merged into each other demonstrating pronounced bleeding. However the inclusion of 2 % PEO into 3 % gelatin conferred for even, consistent printing with little ink bleeding prior to completion of cross linking producing a well-defined grid pattern (Fig. 2b). When using the 3 % gelatin/2 % PEO bioink with 0.1 MPa back pressure, we were able to print a 2 × 2cm^2^ grid with 10 × 10 strut grid. After gelation these constructs demonstrated sufficient strength in that they could be handled. Due to the ability of the 3 % gelatin/2 % PEO hydrogel to form more clearly defined lines by FFF at 37 °C, it was thus designated as a 2-D bioink.

The proliferation and spreading of the hydrogel entrapped cells were observed by monitoring of fluorescence GFP-HEK 293. The hydrogel bioinks were used to deposit and GFP-HUVEC. Using the 3 % gelatin/ 2%PEO hydrogel the GFP fluorescence of HEK 293 was observed spread throughout the gel on day 1 (Fig. 2c). By day 6, detectable GFP-HEK293 fluorescence had dramatically increased (Fig. 2d), with the cells were retained exclusively within the bioink.

The 2-D bioink undergoing crosslinking with 3U/ml mTgase can print for approximately 3mins at 37 °C before the reaction prevents ink flow due to blockage. The amount of HUVEC laden bioink that could be deposited within 3 min via the robotic dispensing system (at 0.1Mpa back pressure) before gelation was assessed. Within this time approximately 900 mm linear length of bioink (of 250 μm width and 25 μm height) was deposited (data not shown).

### Bioink formulation for fabricating a 3D construct at 24 °C

**Fig. 3:**
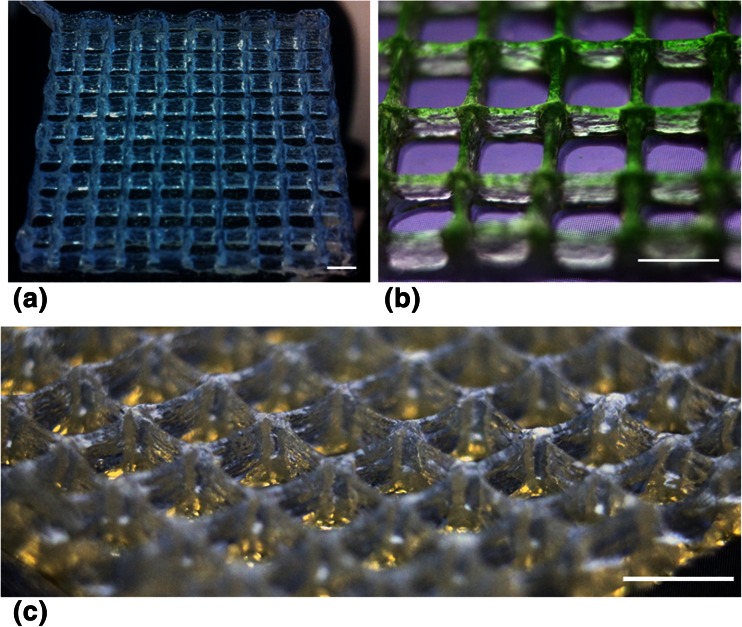
3-D printed gelatin gel woodpile structure viewed at increasing magnification and various perspectives. The 5 % gelatin 3-D bioink was tested for the fabrication of a 3-D woodpile scaffold, number of layers 5 scale bar = 2 mm

Printing hydrogels in 3D requires high viscosity to support construction in the z axis, which could not be met with the 2-D bioink. However, the 5 % gelatin solution could be used to build up the porous structure with dimensions of 1 cm^2^ × 0.5 cm (Fig. 3a, b and c). The temperature of the hydrogel had to be maintained at approximately 24 °C, close to the upper critical solution temperature (UCST) for gelatin, to generate the sufficient viscosity (temperature and rheological properties are characterized below). The “strut like” features are discernible without collapse. The crosslinking proceeded and the resulting scaffold could be stored in water for over a week without dissolving (Figure S2).

### Cell viability within 3D printed gelatin bioinks structures

**Fig. 4:**
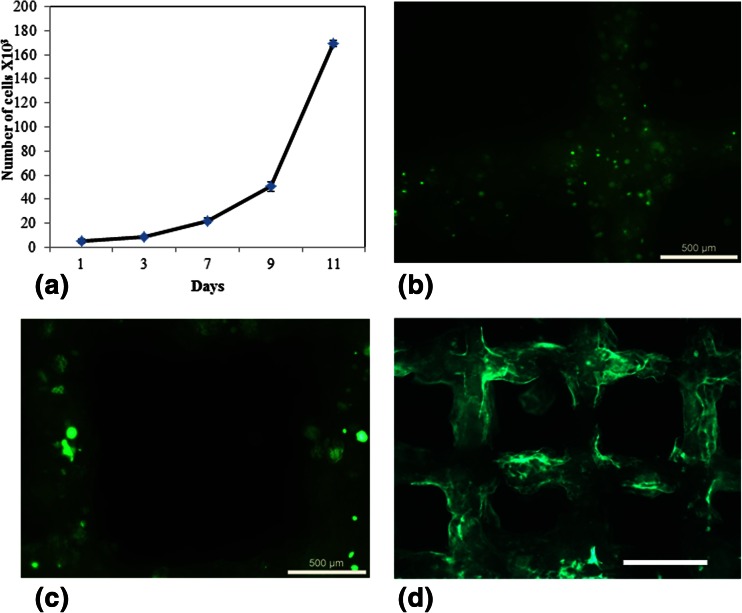
Viability and cell spreading within the 3-D printed hydrogel. Alamar blue quantification of cell viability within a 3-D structure printed with 5 % gelatin bioink containing HEK293 cells (**a**), The GFP fluorescence from HEK293 cells printed on day 2 (**b**), 5 (**c**) and 10 (**d**) demonstrating cytoplasm expansion (**b** and **c**) and spreading through the structure (**d**). Scale bar = 500 μm for (**b**) and (**c**) and 2 mm for (**d**)

The GFP-HEK293 cells were printed into a 5 % gelatin log pile structure of 6 layers high and their viability was monitored by viewing the continuing GFP fluorescence and also quantified by Alamar blue assay. The Alamar blue assay demonstrated that the cells readily proliferate within the gel with the expansion becoming more exponential after 9 days (Fig. 4a).

The GFP fluorescence also demonstrated that the cells were printed intact (Fig. 4b), then the cells were able to spread and form “pseudopodia” like extensions (Fig. 4c) as observed on day 5. By day 10, the cellular proliferation is noticeable by the widespread fluorescence throughout the structure (Fig. 4d).

### Effect of shear stress cell viability with increasing gelatin concentrations

**Fig. 5:**
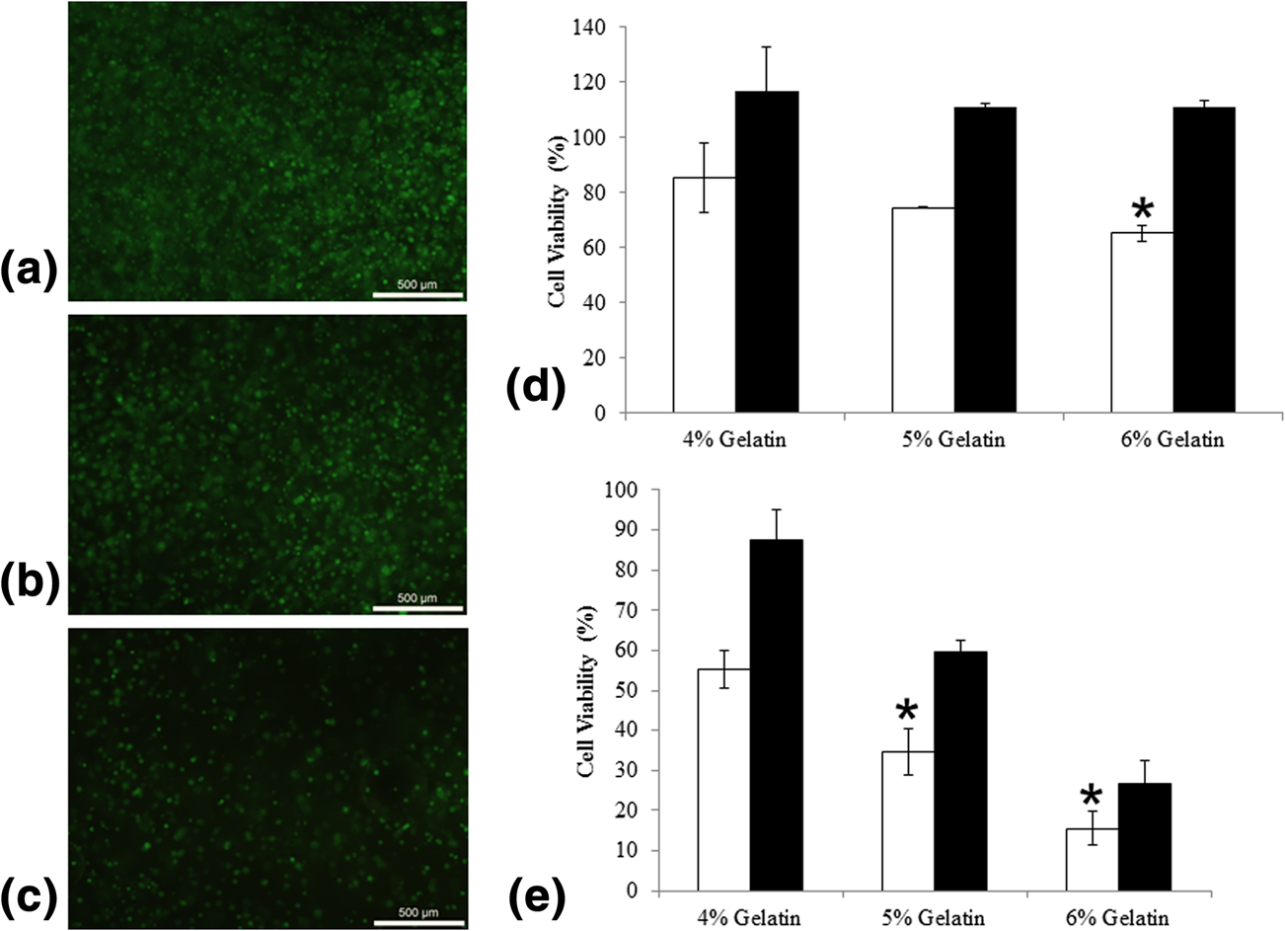
Effect of the deposition shear stress on cell viability. HEK293 cells were robotically deposited at 0.1 MPa back pressure use gelatin concentrations of 4, 5 and 6 % (**a**, **b** and **c** respectively) The viability of HEK293 cells in gelatin without (**d**) and with (**e**) robotic delivery. *Significance demonstrated using *t*-test *p* < 1e^−3^ and *n* = 3

The printing of cells requires their dispensing in bioinks of considerable greater viscosity than that commonly experienced physiologically. Hence the printing may cause excessive shear stress to the cells (Fig. 5). When the cells were initially mixed and dispensed slowly using a 20 ml serological pipette in 4, 5 and 6 % warmed gelatin (at approximately 30 °C), the cell viability post deposition only reduced to 85 % in 4%gelatin (with no significant difference to 5 % gelatin) and to 65 % in 6 % gelatin, the cell populations increased to approximately 110 % of initial density within 48 h (Fig. 5d). However when dispensed by back pressure though the syringe, the disparity in viability between the three gelatin bioink formulations was more obvious. The viability of cells in 4 % gelatin bioink was reduced to 55 % (Fig. 5a), recovering to approximately 87 % by 48 h, in 5 % gelatin bioink dropped to 34 % (Fig. 5b), doubling to approximately 60 % after 48 h and most dramatically in 6 % gelatin bioink the cell viability drops to 15 % (Fig. 5c) and rises to 26 % after 48 h.

The printing of cells in bioink at 24 °C at a rate of 8 mm/cm and back pressure of 0.05 MPa does produce a deleterious shear stress on the cells. However sufficient cells can survive, that go onto proliferate and spread throughout the hydrogel construct. Additives that are known to decrease shear stress related necrosis in other cell systems such as 3 % PEO and 50 % serum were tested, as it will be of future interest to assess any improvement to cell viability with their inclusion. However, these hydrogels could not support 3-D printing and their constructs proved unstable (data not shown). More study is required to improve the cell viability within the printed scaffold.

### Characterization of bioink properties

**Fig. 6:**
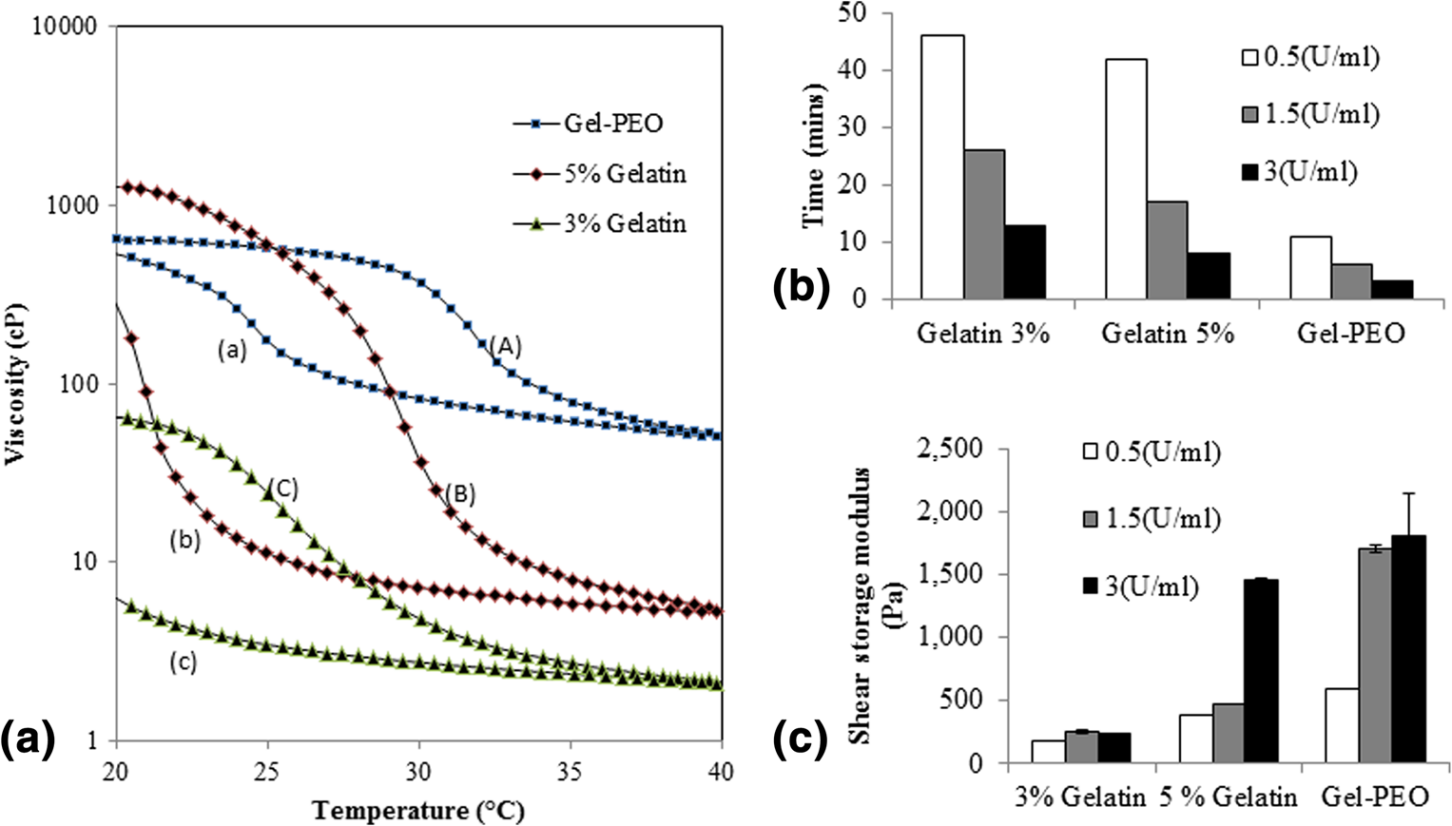
Gelatin Bioink characterization. Heat-cool-heat cycle, change in viscosity with respect to temperature (**a**). (*A*), (*B*) and (*C*) are the heating cycle and (*a*), (*b*) and (*c*) are the cooling cycle. (*A*) and (*a*), (*B*) and (*b*) and (*C*) and (*c*) represent 3 % gelatin/2%PEO, 5 % gelatin and 3 % gelatin solutions respectively. Time taken to initiate gelation as measured by the time required for G′ to overtake G″ (**b**). Gels shear storage modulus at different enzyme concentration. *p* < 1e^−6^ and *n* = 3 (**c**). Test was performed without the addition of mTgase or cells

The uncrosslinked gelatin hydrogels did not demonstrate any significant change in viscosity when the concentration increased from 3 to 5 % gelatin 37 °C (2 to 6cp), however the viscosity of 3 % gelatin/2%PEO was approximately 30 fold greater than the 3 % gelatin-alone preparations at 60cp at 37 °C (Fig. 6). Hence, the addition of the viscous 600K MW PEO helps retain the construct design until gelation starts, this prevents the occurrence of “ink bleeding”, in which the print resolution is lost as the ink spreads following surface contact.

The properties of gelatin solutions are temperature sensitive, therefore bio-ink viscosities were measured with respect to varying temperature, thus the hysteresis caused by heating and cooling cycles were recorded. The viscosity of 5 % gelatin was the most sensitive to temperature changes changed from less than 10cp (at 40 °C) to 1000cp (at 20 °C), furthermore, by having the greatest viscosity at room temperature (24 °C) the 5 % gelatin bioink solution was the most suitable for 3-D printing. Whereas the 3 % gelatin/2%PEO was the least sensitive to temperature changes, allowing it to print at both room temperature to 37 °C (Fig. 6a).

### Enzyme concentration

The time taken to fabricate the constructs shown here were possible within the pre-gelation initiation time window allowed by using 3U/ml mTgase, 3mins for 3 % gelatin/2%PEO and 8 min for 5 % gelatin. However by reducing the enzyme concentration to 1.5U/ml the time widow doubled, with further time extension at 0.5U/ml, this effect will allow the fabrication of larger scaffolds. The enzyme concentration also influenced gelation time of every formulation (Fig. 5b), highlighting this as a possible mechanism for reaction rate regulation. The increase of gelatin concentration had a lesser effect on gelation time. Interestingly, the inclusion of PEO appears to accelerate gelation of 3 % gelatin, for example, from 45 min (3 % gelatin, 0.5U/ml mTgase) to 10 min (3 % gelatin/2 % PEO, 0.5 U/ml mTgase) (Fig. 6b).

The effect of enzyme content on shear modulus was assessed with strength of gels prepared with 0.5U/ml, 1.5U/ml and 3U/ml mTgase (Fig. 5c). For all the bioink preparations, increasing the enzyme concentration conferred for an increase in the modulus (Fig. 6c).

### Porosity of bioink gels

**Fig. 7:**
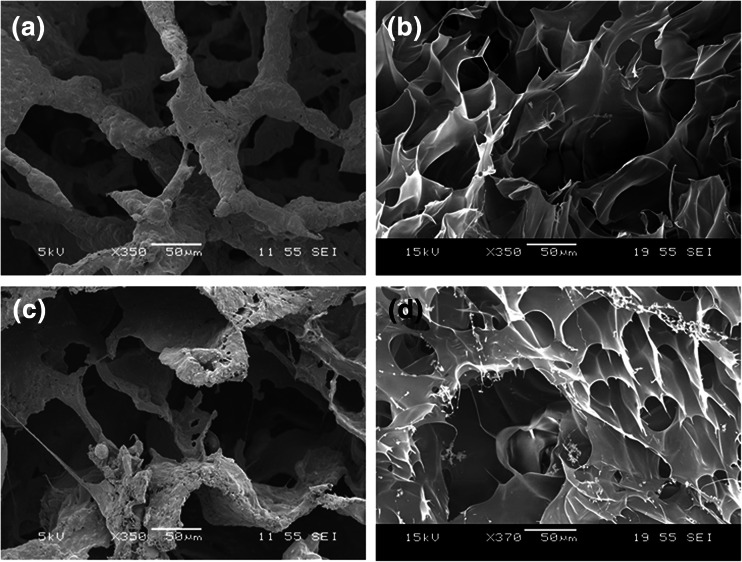
SEM images of lyophilized hydrogels. SEM images of lyophilized 5 % gelatin hydrogel and 3 % gelatin/2 % PEO hydrogels (**a** and **c**) and after dialysis (**b** and **d**)

Hydrogels of 5 % gelatin (Fig. 7a and b) and 3 % gelatin/2 % PEO (Fig. 7c and d) were lyophilized with (Fig. 7b and d) /without (Fig. 7a and c) subsequent dialysis for use in SEM. From the SEM images it appears that the structure within the 3 % gelatin/2%PEO is very similar to that of the 5 % gelatin, hence the inclusion of PEO did not hamper the formation of a protein network. The gel “walls” of the two formulations appeared rough, probably due to the retention of uncrosslinked gelatin and maltodextrin (Fig. 7a and c), dialysis generates a much smoother surface (Fig. 7d). Uncrosslinked maltodextrin has previously been associated with the surface roughness of mTgase crosslinked soy hydrogel and was also found to enhance gel pore size (Chien and Shah 2012; Chambi and Grosso 2006). Various researchers have proposed that hydrogel porosity plays an important role in cell survivability, proliferation and migration (Lien et al. 2009; Mandal and Kundu 2009). The interconnected pores help in cell ingrowth, vascularization and nutrient diffusion for cell survivability. It has been reported that ECM secretion and remodeling increased with increasing pore size within the gelatin hydrogel (Annabi et al. 2010; Workman et al. 2007; Pirlo et al. 2012).

### Extending printing time by mTgase soaking

A key limitation of including the mTgase in the printing bioink is the rate crosslinking, leading to clogging of the printing tip. It was found to be possible for longer sustained printing of 5 % gelatin without mTgase, then crosslinked by soaking in mTgase solution. However these printed structures show profoundly greater disintegration after several days than do the ones printed with the bioink containing transglutaminase (Figure S2). A logical modification of the present system would be to mix the mTgase immediately prior to extrusion so that the bioink in the printer syringe does not clog the system, this would require the design of a more complex print head. Such a mixing system would have to be designed as not to introduce additional cell damaging shear stress.

## Conclusion

The aim of this study was to formulate two bioinks which can entrap and maintain the viability of human cells that can be applied to freeform fabrication system for both the deposition of precise 2-D cell patterns and building up 3-D scaffolds. We have successfully demonstrated that the optimized bioinks allowed the entrapped cells to remain viable and to increase in number for several days.

The HEK293 and HUVECs were selected as the candidate cells for cell entrapment. The HEK293 cells are common for encapsulation studies due to their robustness in such circumstances, as well as their readiness for genetic modification for downstream application of the hydrogel (Lien et al 2009). The hydrogel deposition of HUVECs is also of interest, as the deposition of endothelial cells is studied for printing ordered networks for the promotion of angiogenesis/vasculagenesis (Pirlo et al 2012). It is however very likely that numerous other cell types can be delivered in a similar hydrogel system by FFF due to the native ECM like characteristics of the gelatin hydrogel.

Here, we have established a method of 3-D printing cell bearing gelatin with the utilization of mTgase crosslinking. Future optimization can identify approaches to increase cell viability, increase resolution and also to extend printing time.

As 3-D printing advances in bioengineering, we will look to expanding the applications of the technique. A cellularised, crosslinking gelatin bioink may suit printing constructs for cardiovascular, skin and other soft tissue bioengineering. One interesting application would be to mimic the structure of blood vessels with the delivery of distinct layers of endothelial cells, smooth muscle cells and fibroblast within an ECM like gelatin hydrogel to construct a cellularized vascular prosthesis.

## Electronic supplementary material

Below is the link to the electronic supplementary material.

ESM 1 (MPG 5.25 MB)

Figure S1 HUVEC delivered in bioinks with various gelatin contents. 3 % gelatin/2 % PEO (a), 5 % gelatin (b) and 7 % gelatin (c). Scale bar = 100 μm (a) and 50 μm (b and c) (GIF 258 kb)

(TIFF 280 kb)

Figure S2 Comparison of mTgase crosslinking gelatin methods. Images of FFF 5 % gelatin (containing mTgase) scaffolds built up to 20 layers (a), and 5 % gelatin (without mTgase) scaffolds built up to 20 layers,  then crosslinked by soaking in a mTgase bath (3 % solution) (b). The structures are 20 mm × 20 mm. Colouring dyes are added to the constructs to aid visualization. (GIF 298 kb)

(TIFF 486 kb)

Figure S3 Fluoresence imaging of the FFF 5 % gelatin scaffolds built up to 5 layers, 20 mm × 20 mm × 20 mm. The fluorescent signal from HEK293 cells days 2 weeks after cell seeding. Viewed under stereoscopic microscopy scale bar = 2 mm. (GIF 391 kb)

(TIFF 693 kb)
