# Supplementary figures and images for: Printing cell-laden gelatin constructs by free-form fabrication and enzymatic protein crosslinking
Source: Biomed Microdevices. 2015 Feb 1;17(1):16. doi: 10.1007/s10544-014-9915-8 (PMC4317521; doi:10.1007/s10544-014-9915-8)

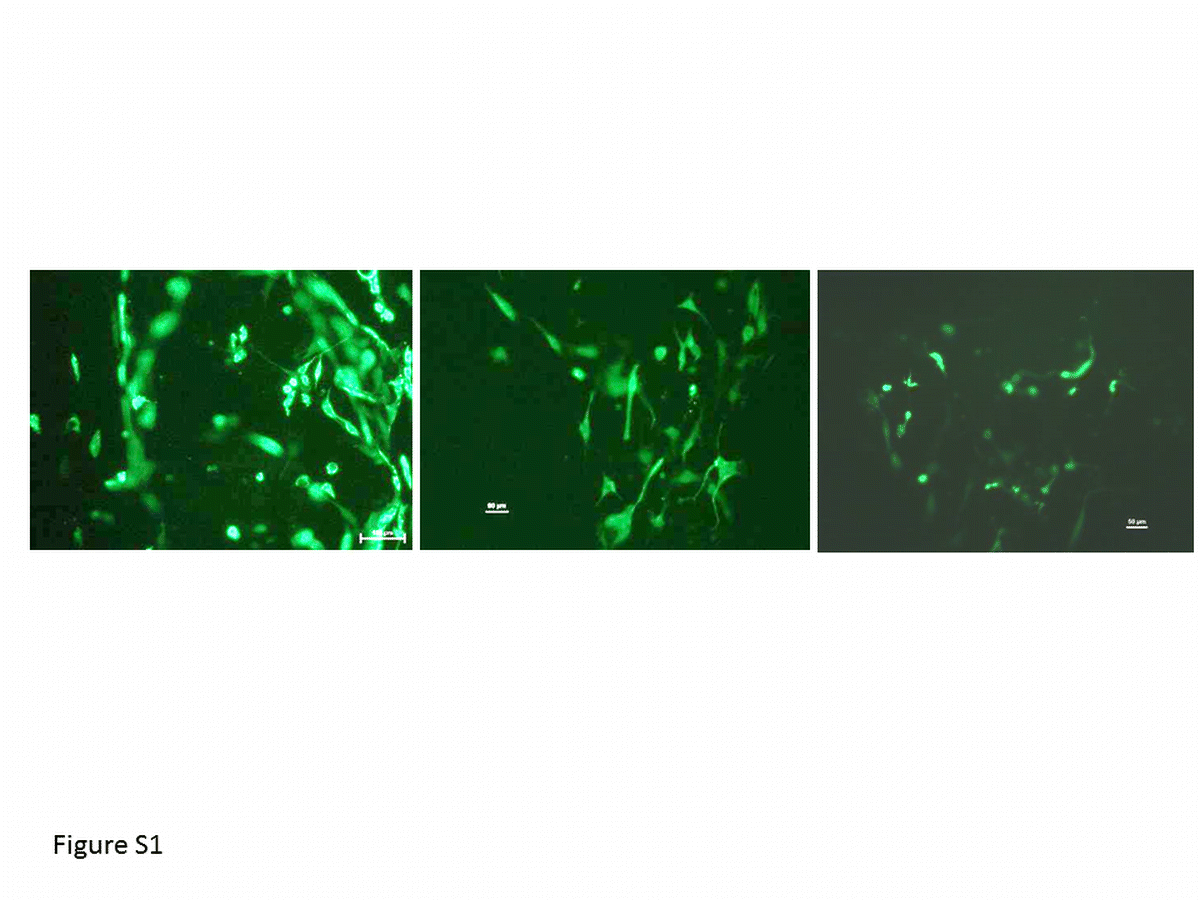

Supplement: Supplementary file 2 — HUVEC delivered in bioinks with various gelatin contents. 3 % gelatin/2 % PEO (a), 5 % gelatin (b) and 7 % gelatin (c). Scale bar = 100 μm (a) and 50 μm (b and c) (GIF 258 kb) [file 10544_2014_9915_Fig8_ESM.gif]

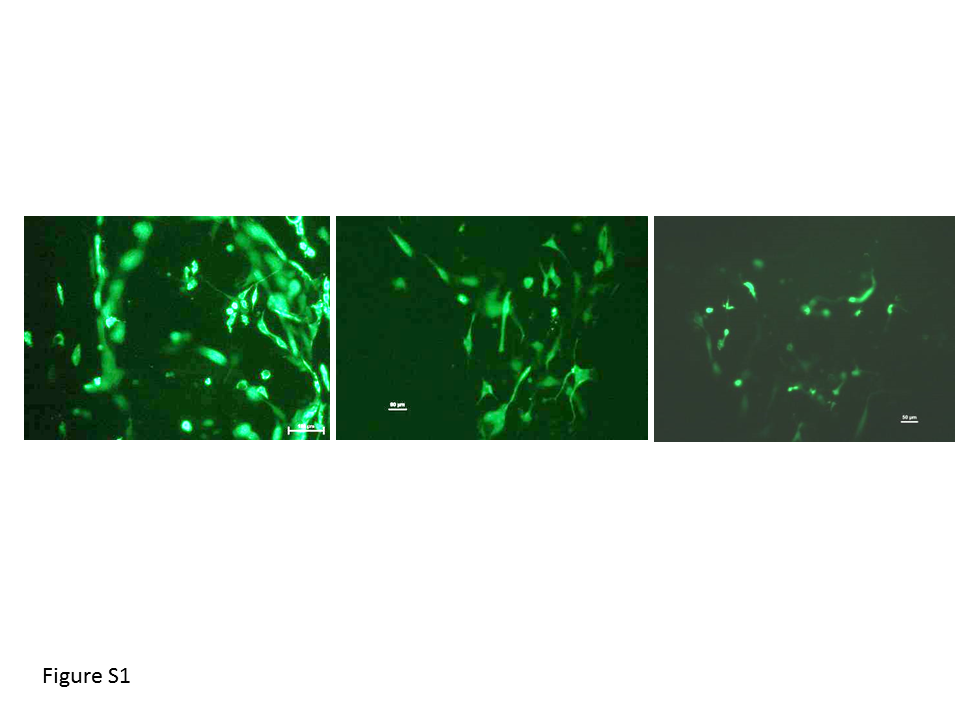

Supplement: Supplementary file 3 — (TIFF 280 kb) [file 10544_2014_9915_MOESM2_ESM.tif]

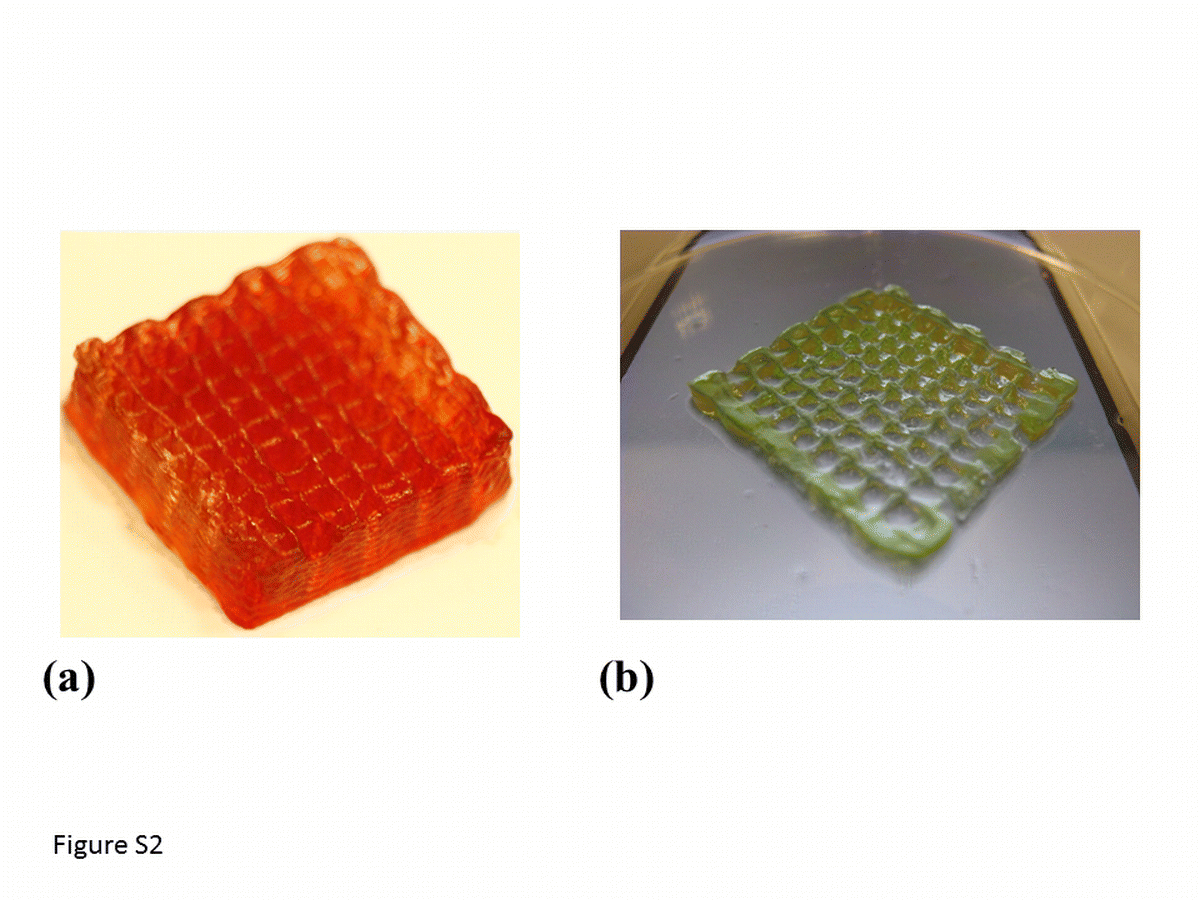

Supplement: Supplementary file 4 — Comparison of mTgase crosslinking gelatin methods. Images of FFF 5 % gelatin (containing mTgase) scaffolds built up to 20 layers (a), and 5 % gelatin (without mTgase) scaffolds built up to 20 layers, then crosslinked by soaking in a mTgase bath (3 % solution) (b). The structures are 20 mm × 20 mm. Colouring dyes are added to the constructs to aid visualization. (GIF 298 kb) [file 10544_2014_9915_Fig9_ESM.gif]

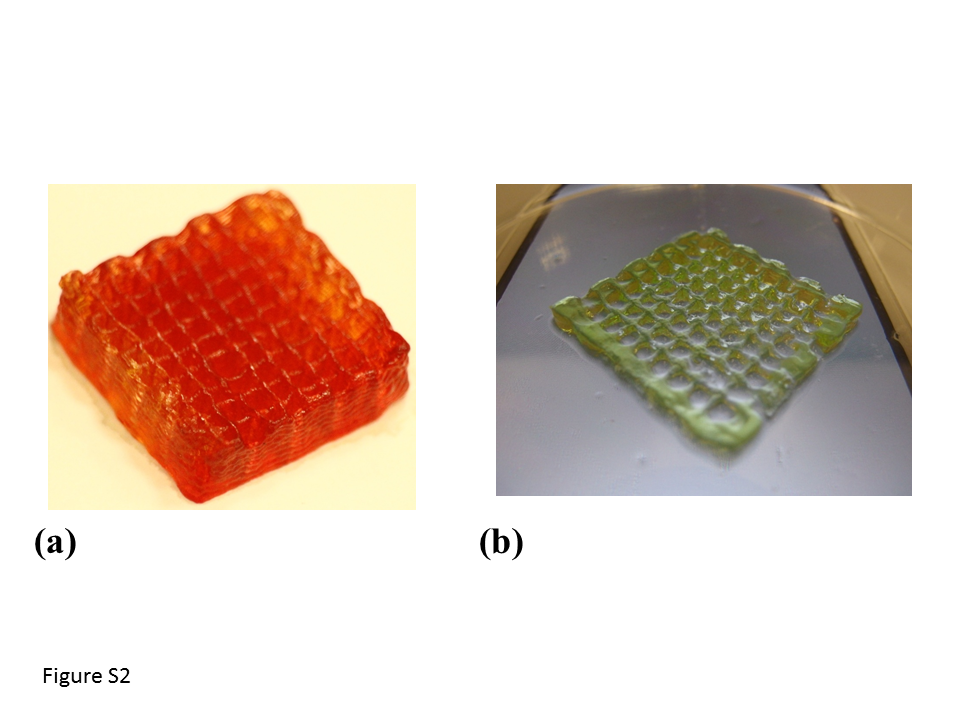

Supplement: Supplementary file 5 — (TIFF 486 kb) [file 10544_2014_9915_MOESM3_ESM.tif]

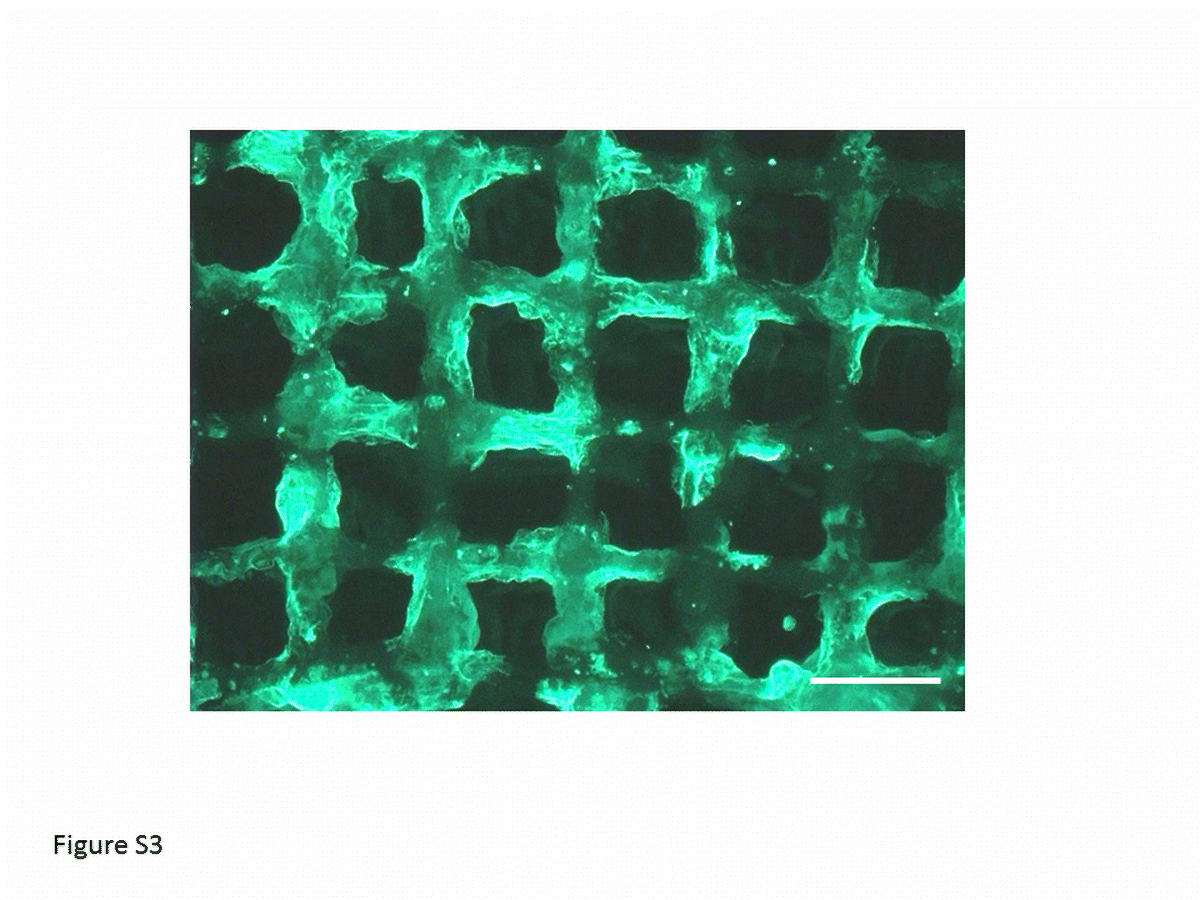

Supplement: Supplementary file 6 — Fluoresence imaging of the FFF 5 % gelatin scaffolds built up to 5 layers, 20 mm × 20 mm × 20 mm. The fluorescent signal from HEK293 cells days 2 weeks after cell seeding. Viewed under stereoscopic microscopy scale bar = 2 mm. (GIF 391 kb) [file 10544_2014_9915_Fig10_ESM.gif]

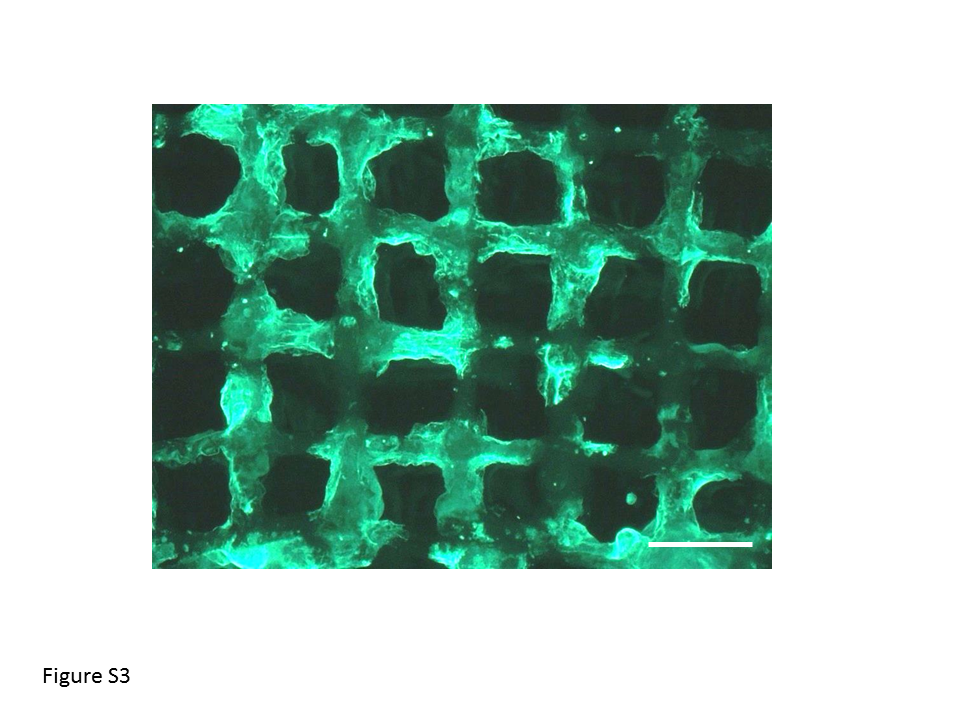

Supplement: Supplementary file 7 — (TIFF 693 kb) [file 10544_2014_9915_MOESM4_ESM.tif]
